# CPL Spectra of Camphor Derivatives in Solution by an Integrated QM/MD Approach

**DOI:** 10.3389/fchem.2020.00584

**Published:** 2020-07-07

**Authors:** Sara Del Galdo, Marco Fusè, Vincenzo Barone

**Affiliations:** SMART Laboratory, Scuola Normale Superiore, Pisa, Italy

**Keywords:** QM/MM, variational/perturbative, vibronic contributions, flexible systems, CPL

## Abstract

We extend a recently proposed computational strategy for the simulation of absorption spectra of semi-rigid molecular systems in condensed phases to the emission spectra of flexible chromophores. As a case study, we have chosen the CPL spectrum of camphor in methanol solution, which shows a well-defined bisignate shape. The first step of our approach is the quantum mechanical computation of reference spectra including vibrational averaging effects and taking bulk solvent effects into account by means of the polarizable continuum model. In the present case, the large amplitude inversion mode is explicitly treated by a numerical approach, whereas the other small-amplitude vibrational modes are taken into account within the harmonic approximation. Next, the snapshots of classical molecular dynamics computations are clusterized and one representative configuration from each cluster is used to compute a reference spectrum. In the present case, different clusters correspond to the two stable conformers of camphor in the S1 excited electronic state and, for each of them, to different numbers of strong solute-solvent hydrogen bonds. Finally, local fluctuation effects within each cluster are taken into account by means of the perturbed matrix model. The overall procedure leads to good agreement with experiment for absorption and emission spectra together with their chiral counterparts, thus allowing to analyze the role of different effects (stereo-electronic, vibrational, environmental) in tuning the overall experimental spectra.

## 1. Introduction

Circularly polarized luminescence (CPL) and electronic circular dichroism (ECD) are complementary techniques giving access to the properties of the excited and ground electronic states, respectively for chiral chromophores or chromophores embedded in chiral environments (Longhi et al., 2016; Tanaka et al., 2018). However, the magnetic dipole transition moment is generally much smaller than the electric one, thus hindering the investigation of systems showing strong luminescence (Riehl and Muller, 2012). Therefore, for both chiroptical spectroscopies, electric dipole forbidden and magnetic dipole allowed transitions represent a particularly appealing situation.

Camphor and its derivatives, and in particular their formally forbidden *n* → π^*^ carbonyl transitions, have been extensively studied by chiroptical methods, from both experimental and computational points of views (Dekkers and Closs, 1976; Schippers et al., 1983; Pritchard and Autschbach, 2010; Longhi et al., 2013; McAlexander and Crawford, 2015; Duong and Fujiki, 2017). Nevertheless, simulation of the camphor CPL is far from being straightforward because of (i) the inherent dependence of the vibrational information from solvation interactions and (ii) the bisignate nature of the spectra. This latter phenomenon was recently qualitatively investigated by Longhi et al. (2013) and Duong and Fujiki (2017). According to Kasha's rule (Kasha, 1950), luminescence is expected to occur from the lowest excited electronic state. Therefore, within the Franck-Condon approximation, the first ECD and CPL transitions should have the same sign. In fact, the bisignate shape is a consequence of the pyramidalization of the carbonyl moiety, which gives rise to two low-energy conformers connected through a large amplitude motion (LAM) in the first excited state. This interconversion path needs to be properly accounted in order to reproduce and evaluate the different vibronic contributions in the spectra (Cerezo et al., 2016; Baiardi et al., 2017).

In this contribution, we apply a QM/MD approach to perform accurate simulations of camphor one-photon absorption (OPA) and emission (OPE) spectra and of their chiral counterparts ECD and CPL. We employ our recently developed ONIOM/EE-PMM (Del Galdo et al., 2019, 2020) procedure to model solvent effects on the electronic properties of the chromophore. The method belongs to the general category of multiscale procedures and merges variational and perturbative approaches in the calculation of spectroscopic features. Starting from extensive classical simulations of a target electronic state of a chromophore, effective clustering procedures are applied in order to identify a relevant set of sub-trajectories within the complete sampling. Within each cluster, one reference snapshot is utilized for an ONIOM calculation performed within the Electronic Embedding framework [ONIOM/EE, i.e., the variational approach (Dapprich et al., 1999; Vreven et al., 2006; Chung et al., 2015)]. Then, the local fluctuations within each sub-trajectory are treated via the Perturbed Matrix Method [PMM, i.e., the perturbative approach (Aschi et al., 2001; Del Galdo et al., 2018; Zanetti-Polzi et al., 2018)]. The procedure permits to strongly reduce the computational costs when compared to more conventional QM/MM approaches, without lowering the accuracy of the final results.

On top of this, the vibrational contributions to the final spectra are also modeled. We exploit for the purpose a general computational tool that allows the simulation of different kinds of one-photon spectroscopies and supports inclusion of mode-mixings, as well as Franck-Condon (FC) and Herzberg-Teller (HT) effects. Finally, internal coordinates are used in order to minimize the coupling between normal modes and LAMs, which are properly accounted for and treated separately.

## 2. Methods

### 2.1. Combining QM and MM Methods, Step 1: Sampling Solute Internal Motions and Solvent Effects

**Figure 1:**
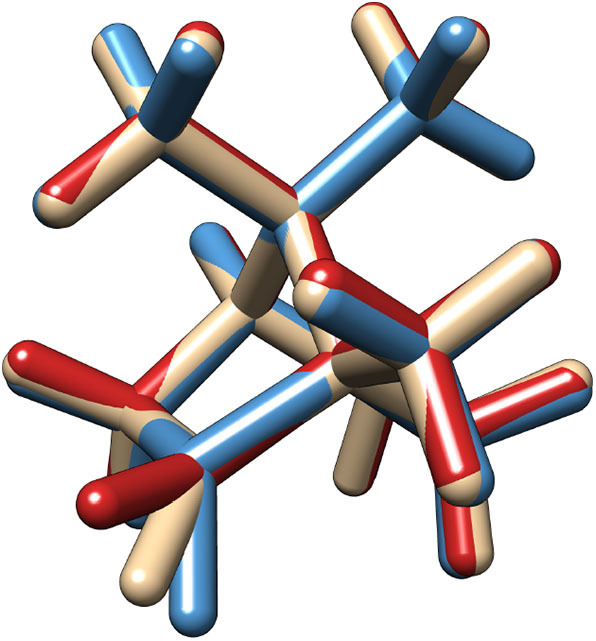
Superimposed structures of the two most stable conformers (Conf.1 in blue and Conf.2 in red) and of the transition state between them in the first excite state.

Molecular Dynamics simulations of the ground and first excited electronic states of camphor in methanol were employed to generate the ensembles needed to evaluate the solvatochromic shifts on absorption and emission spectra. In view of the structural rigidity characterizing the camphor ground state, the corresponding MD simulation was run by constraining the solute at its equilibrium geometry. In this way, we did not take into account internal motions through classical approaches, thus adding their contribution *a posteriori* by means of full QM calculations of vibrational modulation effects by means of models based on the Franck-Condon principle (see sections 2.3). Conversely, as already reported (Longhi et al., 2013; Duong and Fujiki, 2017), in its first electronic state camphor undergoes an internal deformation associated to the carbonyl out of plane bending. After the characterization of the LAM (*vide infra*), we accounted for its effects by extracting the most representative conformers characterizing the excited state potential energy surface as a function of the carbonyl out of plane bending (namely the two minima of the curve reported in Figure 1). Then, both conformers were utilized for the classical MD sampling performed by constraining the solute geometry while the solvent molecules followed their unconstrained motion. The other internal degrees of freedom of the solute were taken into account in a separate step by full QM computations of vibronic effects within the harmonic approximation. For each MD ensemble the corresponding emission spectra were computed, thus obtaining the final spectra by proper weighting of the results. In Table 1, the relative stabilities of the two conformers are reported, which were used to compute the respective Boltzmann population at 300 K. Remembering that the solute was kept rigid during the MD runs for both ground and excited electronic states, the only properties differentiating the two simulation ensembles were the geometry of the solute and the atomic charges, the latter possibly including virtual sites (VSs) to represent oxygen lone pairs. The positions of VSs were determined by first locating the O-VS distance and the C-O-VS angle for the centroids of localized molecular orbitals at the sp2 oxygen atom of formaldehyde and propanone by using the Boys localization procedure, as reported in Macchiagodena et al. (2016) and Del Galdo et al. (2020). By averaging the (nearly identical) results, we obtained a value of 0.3067 Å for the distance and 111.3 degree for the angle. For the ground electronic state we imposed that the VSs, the O, the C and the other two atoms bound to C (i.e., the two C atoms) lie on the same plane (O-C(O)-C plane). Instead, for the excited state the VSs were constrained in the plane orthogonal to the plane defined by the normal to the C-C-C plane and oxygen atom. Separation of the charge between the oxygen nucleus and the VSs (having vanishing masses) was achieved by imposing that both VSs have the same charge and reproducing the classical dipole moment of the molecule in the target electronic state. The role of VSs was evaluated by performing two MD simulations (with and without their inclusion) for the ground electronic state. Specific solute-solvent interactions were analyzed by computing the probability of hydrogen bonding (HB) between the carbonyl oxygen and the hydroxyl group of methanol with the help of the so-called F function (Pagliai et al., 2003; Del Galdo et al., 2019, 2020), based on exponential decays for deviations from both radial and angular optimal values obtained from pure methanol simulations (we obtained 1.8 Å and 6 degrees for the optimum distance and angle, respectively and 0.2 Å and 5 degrees for the corresponding half width at half maximum, HWHM).

**Table 1:** Relative Electronic Energy (ΔE), Free Energy at 300 K (ΔG), Free Energy at 300 K in Solution (ΔG PCM) Values (kJ mol^−1^) for the first excited state. Electronic energies computed at Cis(d) B2 levels of theory, vibrational and PCM correction computed at TD-DFT B3 level of theory^*a*^.

	**ΔE**	**ΔG**	**ΔG PCM**	**Pop.^***a***^**
Conf. 1	0.00	0.00	0.00	96.97
Conf. 2	8.24	8.48	8.73	2.93
TS	15.37	16.19	17.00	0.10

a *Percent population factors (Pop., computed from the Boltzmann distribution) based on ΔG PCM*.

### 2.2. Combining QM and MM Methods, Step 2: The ONIOM/EE-PMM Method

Solvatochromic shifts on the different spectra of camphor were evaluated by the ONIOM/EE-PMM procedure we recently proposed (Del Galdo et al., 2019, 2020). The procedure merges variational and perturbative approaches to get a cheap yet accurate characterization of the perturbing effects exerted by an embedding environment on the quantum mechanical properties of a chromophore. The first step is the identification of a set of basins/clusters within a production MD run. For each sub-sampling, a reference frame is chosen as the most representative one of the corresponding cluster and its spectrum is evaluated by a variational procedure (ONIOM/EE) in which the QM model system (here the Camphor molecule) is embedded in a set of point charges representing the environment (here the methanol molecules) (Vreven et al., 2006). For all the other frames of each cluster, the fluctuations of environmental effects with respect to the reference configuration are computed *a posteriori* by a perturbative approach (PMM). In details, for each frame of each sub-trajectory, the system Hamiltonian is written as the diagonal matrix of the eigenvalues of the reference configuration plus a perturbation matrix representing the difference of the electrostatic potential between the considered frame and the reference value. Diagonalization of this matrix provides a set of eigenvalues (electronic states) representing the instantaneous effects of the embedding environment as provided by the MD trajectories. The operator representing the variation between the perturbing effects exerted by the environment in each frame and the reference, is modeled by exploiting the latest development of the PMM procedure, that is by expanding the perturbing electrostatic potential within the atomic region around each atomic center (atom-based expansion) (Zanetti-Polzi et al., 2018). Interested readers can find details of the procedure in Aschi et al. (2001), Zanetti-Polzi et al. (2018), Del Galdo et al. (2019), and Del Galdo et al. (2020) while specific computational details are reported in the following subsections.

### 2.3. Combining ONIOM/EE-PMM and Vibronic Calculation

The fist step is the simulation of the vibrational modulation (vibronic) effects in electronic spectra using models based on the Franck-Condon principle and a continuum description (PCM) of bulk solvent effects, as described in detail in Barone et al. (2009), Bloino et al. (2010), and Bloino et al. (2016). In particular, we employed the so-called time-independent (TI) approach, in which the band-shape is obtained as the sum of the individual transitions between the vibrational states of the initial and final electronic states. In order to best decouple the effect of the LAM, we employed the Vertical Gradient (VG) model (Baiardi et al., 2016), which requires the vibrational frequencies of the initial state, but only the forces of the final state at the equilibrium geometry of the initial one (Barone et al., 2009; Bloino et al., 2010, 2016).

Once computed the vibronic spectra for the reference structure of each conformer, they need to be combined with the outcome of the ONIOM/EE-PMM procedure to obtain the final spectra.

At the FC level, a simple shift by the transition energy and scaling by the transition moments for each snapshot leads, after proper averaging, to the final spectrum. Furthermore, on the grounds of previous experience (Del Galdo et al., 2020), effects related to environment fluctuations were considered negligible for magnetic transition dipole moments.

Inclusion of HT terms, accounting for the dependence of the transition dipole moments on the nuclei position, would require the computation of the derivatives of the perturbed transition dipole moments for each MD snapshot. Since this model would become too computationally demanding, we assumed, as in previous works (Del Galdo et al., 2020), that the difference between FC and FCHT spectra is explicitly evaluated only for the reference configuration, whereas the effect of solvent fluctuations is taken as half the value explicitly computed for the FC part as described above. In the following sections, the FCHT acronym will be used to indicate the inclusion of both FC and HT terms in the calculations.

### 2.4. Computational Details

All geometry optimizations and frequency calculations were carried out with a development version of the Gaussian suite of quantum chemical programs (Frisch et al., 2019), whereas all the MD simulations were performed with the GROMACS software (Berendsen et al., 1995). All the MD production runs of ground and first excited electronic states were performed by constraining the chromophore in the corresponding equilibrium geometry as obtained in the framework of the Density Functional Theory (DFT) (Lee et al., 1988) and its Time Dependent extension (TD-DFT) with the hybrid B3LYP functional (Becke, 1988; Lee et al., 1988) with additional empirical dispersion contributions (D3BJ) (Grimme et al., 2011) and the jul-cc-pVDZ (Dunning, 1989; Papajak et al., 2011) basis set (B3). For each simulation, the solute atomic charges were computed in vacuum at the proper equilibrium geometries by means of the CM5 model (Marenich et al., 2012) at the same level of theory. As mentioned above, two simulations of the ground state were performed including or not virtual sites on the oxygen atom to model directional effects related to sp2 lone pairs. Only the model including virtual sites was used for the simulations of the excited electronic state. All atom Lennard-Jones parameters were taken from the OPLS force field (Jorgensen et al., 1996) MD simulations under periodic boundary conditions were performed in the isothermal-isochoric ensemble (NVT) in order to avoid the additional parameters related to isobaric thermostats required by NPT simulations. The integration step was 2 fs and the temperature was kept constant (300 K) by the velocity-rescaling (Bussi et al., 2007) temperature coupling. Hard geometrical parameters were constrained using the LINCS algorithm (Hess et al., 1997). The particle mesh Ewald method (Darden et al., 1997) was used to compute long range interactions with grid search and cut-off radii of 1.1 nm. A cubic simulation box was utilized and the density was calibrated to obtain in the NVT MD simulations a pressure equal, within the noise, to that provided by a corresponding simulation of the pure solvent. The latter was carried out in the NVT ensemble at *T* = 300 K and imposing a box density equal to the experimental density of pure methanol at standard conditions (24.58 mol/l, Goodwin, 1987). All the production runs were 10 ns long. We analyzed the solvent effects within each simulation, by computing the probability of the carbonyl oxygen to be engaged in hydrogen bonds with methanol molecules as outlined above (that is, by means of the F function). For each production run, we partitioned the sampling into 4 clusters characterized by two, one (with either VS) or none solute-solvent hydrogen bond, respectively. The partitioning was performed using a threshold of 0.7 for the presence of an effective hydrogen bond. Next, for each cluster, the reference configuration used for the ONIOM/EE calculations is taken as a “collective frame” representative of the average configuration of the molecular environment for the corresponding cluster. To this aim, we extracted 30 snapshots sequentially from the trajectory, from each snapshot we cut a sphere of 30 Å centered around the solute and we assembled them into a collective configuration and we assigned to each environmental atom 1/30 of the actual atomic charge. The first 11 electronic states and the complete matrix of the corresponding dipole moments were computed for the 4 reference configurations exploiting the ONIOM/EE model with the QM part described at the TD-DFT/CAM-B3LYP (Yanai et al., 2004) level in conjunction with the jul-cc-pVDZ (Dunning, 1989; Papajak et al., 2011) basis set. For each electronic state, the corresponding atomic charges were also computed at the same computational level using the CM5 recipe. The above results were then utilized to apply the PMM approach for evaluating fluctuation effects within each cluster. Free energies were determined by adding to electronic energies zero-point energy, thermal, and PCM solvation contributions evaluated in the framework of the rigid rotor/harmonic oscillator approximation (Bloino et al., 2012; Cappelli et al., 2012; Mennucci, 2012). Transition energies have been corrected by the differences between TDDFT/CAMB3LYP/jul-cc-pVDZ and Cis(d) B2PLYPD3/jun-cc-pVTZ (Grimme and Neese, 2007; Jacquemin et al., 2009; Ottochian et al., 2020) (B2) results for isolated camphor. The LAM associated with the double-well potential has been characterized in the framework of the ICPH-model (Baiardi et al., 2017), using 251 DVR basis functions and a step size of 4°. Vibronic spectra were simulated starting from the structure and force constants computed taking into account solvent effects by PCM (Cappelli et al., 2012; Mennucci, 2012). OPE and CPL spectra were computed within the so-called time-independent (TI) approach, employing VG|FC and and VG|FCHT models. The default Gaussian parameters of the class-based integral prescreening scheme were employed (${\text{C}}_{1}^{max}$ = 50, ${\text{C}}_{2}^{max}$= 30, ${\text{N}}_{I}^{max}$ = 10^9^). Normal modes connected with the LAM or methyl rotations were removed. Gaussian distribution functions with HWHM of 900, 550, and 450 cm^−1^ were used as broadening functions for pure electronic ONIOM/EE-PMM, vibronic PCM, and combined ONIOM/EE-PMM vibronic approaches, respectively. Spatial Distribution Functions plotted as isosurfaces and virtual sites were visualized employing the Caffeine software (Salvadori et al., 2016; Lazzari et al., 2020).

## 3. Results

### 3.1. Characterization of the LAM

**Figure 2:**
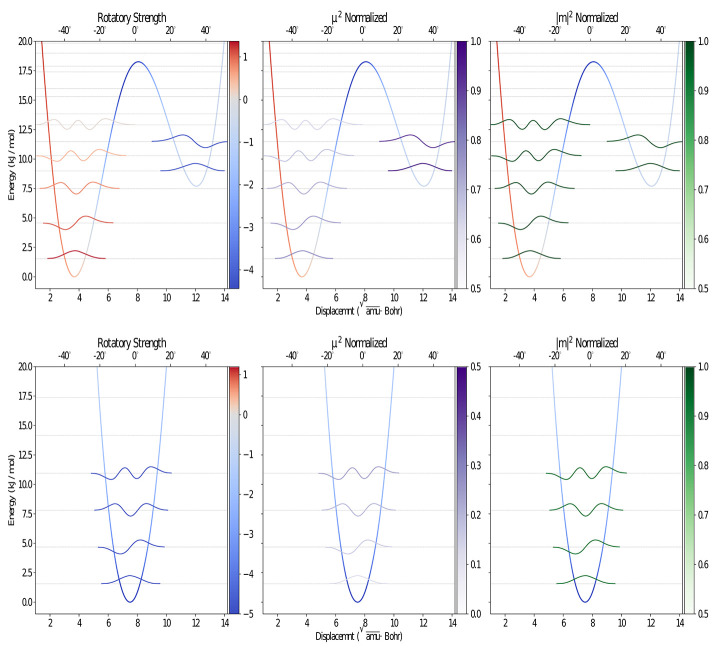
Graphical representation of the PES along the CO out-of-plane bending for the S_1_ **(upper)** and S_0_ **(lower)** electronic states, computed at the B3LYP/Jul-cc-pVDZ (D3BJ) level of theory using the ICPH framework. The vibrational levels and wave functions with contribution above 0.5% at 300K, computed using the variational DVR-based approach, are also reported.

As already mentioned in the introduction, the lowest band in the camphor absorption spectrum is associated with the formally forbidden *n* → π^*^ carbonyl transition. However, experiments reveal the presence of a bisignate band in the CPL spectrum that can be connected with a breakdown of the Frank-Condon regime or with the involvement of more than one electronic state in the emission process. Since the second transition lies at a significantly higher energy than the first one, this behavior can be attributed, as previously suggested, to the flexibility of the system. Indeed, in its first electronic state camphor undergoes an internal deformation associated to the carbonyl out of plane bending: a LAM connects two minima through a flat transition state (see Figure 1). These kinds of LAMs are generally ill described by harmonic models (Baiardi et al., 2015, 2016), but use of internal coordinates strongly reduces the coupling of the LAM from the other modes, leading, to a good approximation, to an effective one-dimensional problem (Cerezo et al., 2016; Baiardi et al., 2017). The PES associated with the one-dimensional LAM was explicitly computed through a scan along the CO out-of-plane (OOP) coordinate and it was used to solve numerically the vibrational Schrödiger equation with the help of a quasi-variational approach employing the discrete variable method (Light et al., 1985; Bačić and Light, 1989; Colbert and Miller, 1992; Light and Carrington, 2000; Baiardi et al., 2017). In the upper panels of Figure 2 the PES of the first excited state along the LAM is reported together with the associated vibrational levels obtained with the DVR method, whereas in the bottom panels the ground state PES computed along the same path is reported. Moreover, the vibrational wave functions of those states relevant at 300 K (population above 0.5%) are drawn for both S1 and S0. The different colors highlight the dependence on the LAM of the electric and magnetic transition dipole moment and the angle between them. As expected, in the ground state only one conformer, corresponding to a flat CO structure, is observed along the LAM. On the other hand, the results of the excited state reveal the presence of two conformers corresponding to the out-of-plane bending of the CO, which are separated by a relatively high (18 kJ/mol) transition state (Conf. 1 and Conf. 2). It should also be noted that in S1 the accessible vibrational states at 300 K are well-localized and separated into the two wells. As observed before (Longhi et al., 2013; Duong and Fujiki, 2017), the sign of CPL shows a strong dependence on the LAM. However, as it can be seen in the panels of Figure 2, the most relevant variations are connected with the relative orientation between the two transition dipoles and with the magnitude of the electric transition dipole moment, whereas the corresponding magnetic moment is only negligibly affected by the LAM. Vibronic contributions of the other modes were then described by the Vertical Gradient (VG) model, neglecting the contribution of the LAM already accounted for explicitly. This approach is based on the assumption that the other modes rearrange faster than the LAM (Stendardo et al., 2012; Cerezo et al., 2016; Baiardi et al., 2017).

Finally, the strong dependence of the electric transition dipole moments on the nuclear position was accounted for by including Herzberg-Teller (HT) contributions.

### 3.2. Molecular Dynamics Results

**Figure 3:**
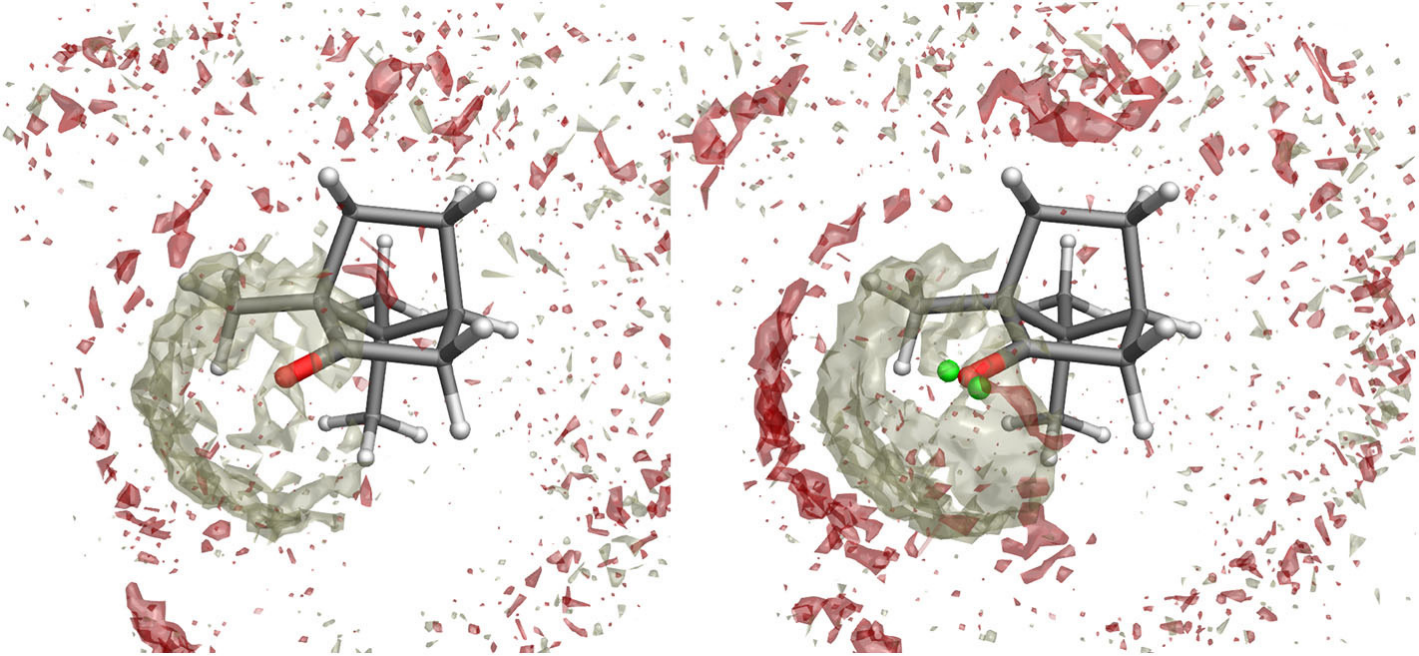
Spatial Distribution Function of methanol molecules around camphor from the ground state simulation when the standard atomic charges **(left)** or the VSs representation **(right)** are employed. Density distributions of (hydroxyl) hydrogen and oxygen atoms in methanol are represented as white and red volumes, respectively.

**Figure 4:**
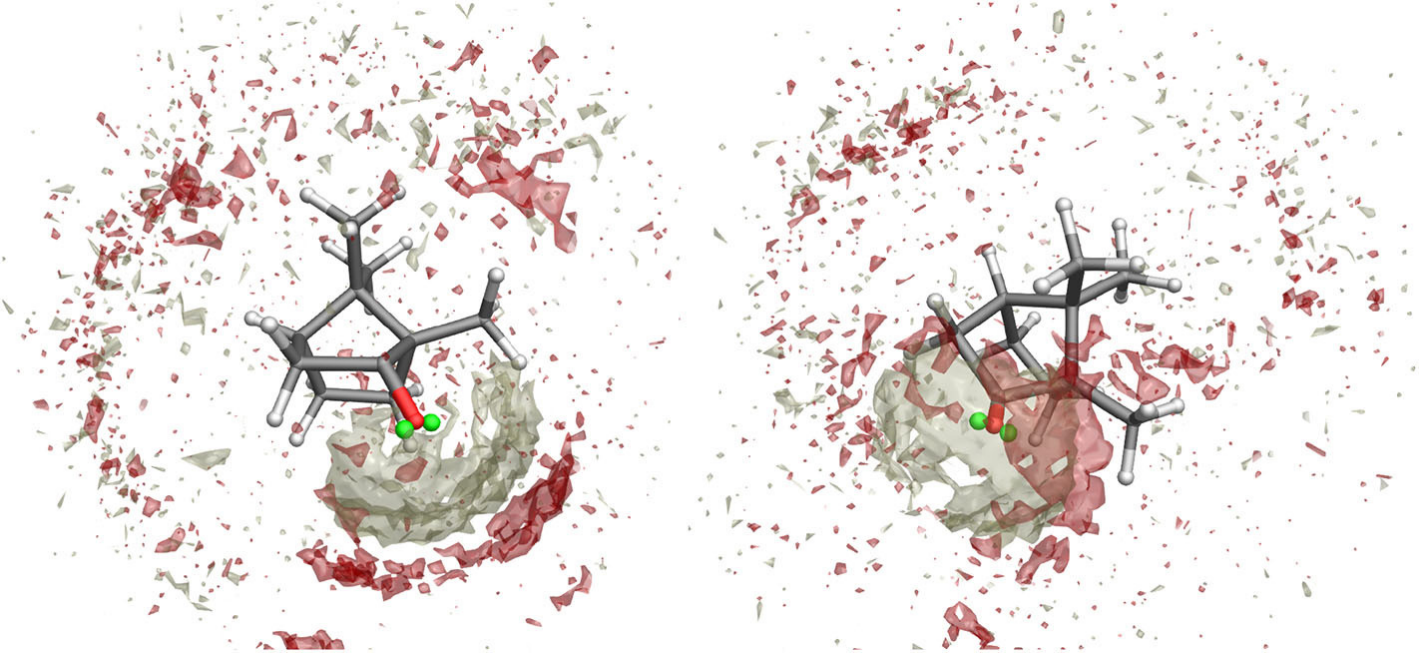
Spatial Distribution Function of methanol molecules around camphor from the first excited state simulation of Conf. 1 **(left)** and 2 **(right)**. Density distributions of (hydroxyl) hydrogen and oxygen atoms in methanol are represented as white and red volumes, respectively.

**Figure 5:**
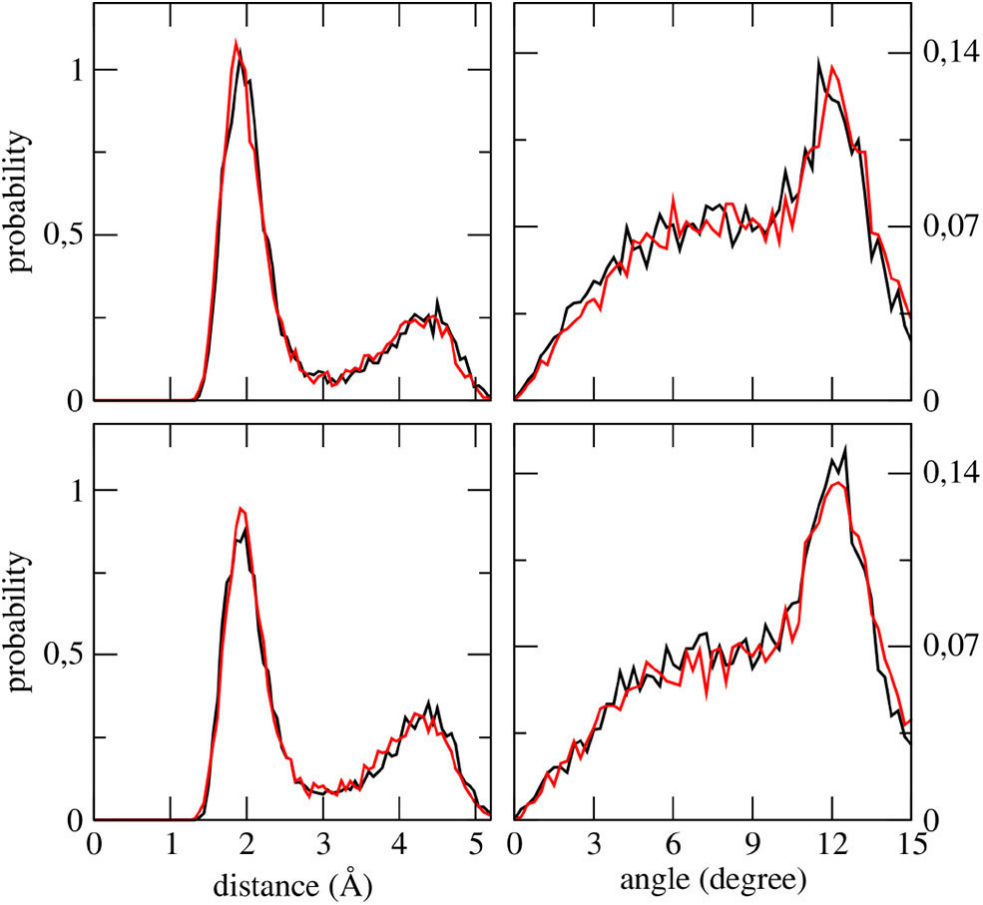
Distribution functions of each VS—(methanol hydroxyl) H distance **(left)** and of each VS—(methanol hydroxyl) H—(methanol hydroxyl) O angles **(right)** as obtained from the first excited state simulation of camphor in methanol. Upper panels refer to the Conf. 1, lower panels refer to Conf. 2. For all the panels, data computed for the first and second VSs are shown as black and red lines, respectively.

**Figure 6:**
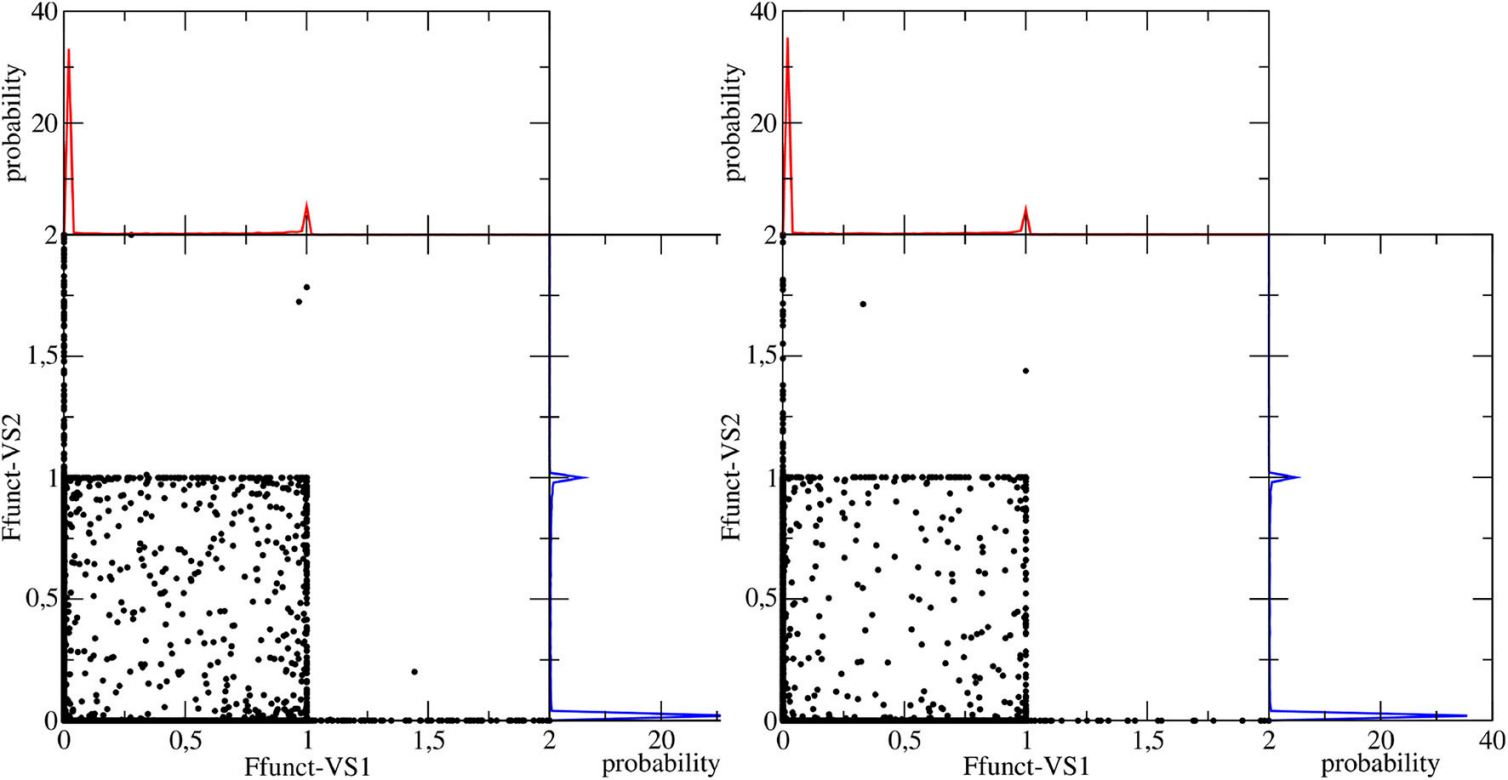
Space defined by the values of the F function computed for the each VS in the camphor molecule. Left and right panels correspond to Conf. 1 and Conf. 2, respectively. In each panel the corresponding probability distributions of each F function are also shown.

The effectiveness of the interaction between camphor and the embedding solvent was at first assessed by computing the Spatial Distribution Function (SDF) of the methanol molecules around the solute for the ground state simulation. In Figure 3, we compare the results of the SDF calculation performed on simulations involving or not VSs. More precisely, we computed the density distributions of (hydroxyl) hydrogen and oxygen atoms in methanol thus addressing the SDF of the partners of probable HB with camphor oxygen. It is apparent that inclusion of VSs increased the (average) number of methanol molecules around oxygen atom. Moreover, these results show that the arrangement of the solvent is slightly asymmetrical, with a more pronounced effect when the VSs are employed. As a consequence (i) we utilized this simulation as the statistical ensemble to evaluate the solvent effects on the absorption spectra and (ii) also for the production MD runs of the (first) excited electronic state we modeled the camphor including VSs. In Figure 4, the SDFs corresponding to the MD runs of the two conformers simulated to account for the first excited state of camphor are reported. The results suggest that the camphor oxygen (both in the ground and excited states) is engaged in HBs with the solvent molecules. In the following, for the sake of brevity, only the results obtained from the excited state simulations are reported while those corresponding to the ground state simulation can be found in the Supplementary Material (see Figures S1, S2). Nevertheless, it is worth noting that very similar results were found from the classical sampling. We computed the distribution of (i) the distance between each VS and the closest methanol hydroxyl hydrogen and (ii) the angle these two form with the oxygen in methanol. The results obtained from both the excited state simulations are reported in Figure 5. Nearly identical profiles are obtained for the two conformers and, within a given conformer, for each VS: while a clear peak appears in the radial distributions, a less pronounced one characterizes the angular distributions thus suggesting that some geometrical hindrance might weaken HB interactions. Next, we analyzed the HB probability for each excited state simulation by computing the F function described in the section 2. The results reported in Figure 6 show that for both conformers, most of the classical sampling is characterized by the absence of any significant HB. However, we also obtained a non-negligible probability of having one HB for each VS. According to our computations, each methanol molecule was considered to interact with either one VS or the other, thus implying that a probability of around 1 for both the interaction sites corresponds to a total number of 2 HBs for the molecule. On the basis of these results, we decided to partition both the excited state trajectories into a set of clusters, each characterized by a different number of HBs occurring between the camphor and the solvent. As shown in Table 2, the largest cluster accounts for the absence of any significant HB (noHB), then we identified two clusters characterized by one HB depending on the VS involved in the interaction (HB-VS1 and HB-VS2) and the fourth cluster includes the remaining snapshots where at least 2 HBs occur (twoHB). As mentioned above, very similar results were found when the ground state simulation was analyzed, thus allowing us to apply the same partitioning procedure to cluster the ground state trajectory (see Table S1 for the results).

**Table 2:** Population per cent of the clusters within the excited state simulation of Conf. 1 and 2.

	**Conf. 1**	**Conf. 2**
noHB	58.04	65.01
HB-VS1	18.79	17.35
HB-VS2	21.58	16.9
twoHB	1.59	0.74

The partitioning is the first step toward application of the ONIOM/EE-PMM method, with the four clusters providing the different statistical ensembles from which the representative frames to be employed for the ONIOM/EE calculations were extracted. Then, the PMM procedure was applied to account for fluctuations (within each cluster) of the external electric potential tuning the electronic properties of the chromophore.

### 3.3. Spectroscopic Results

In order to increase the accuracy of the results, all the transition energies were shifted with reference to the values computed in the gas-phase at the Cis(d) B2PLYPD3/jun-cc-pVTZ level, which is significantly more reliable than TD-DFT with hybrid functionals (Grimme and Neese, 2007; Jacquemin et al., 2009; Laurent and Jacquemin, 2013; Ottochian et al., 2020).

**Figure 7:**
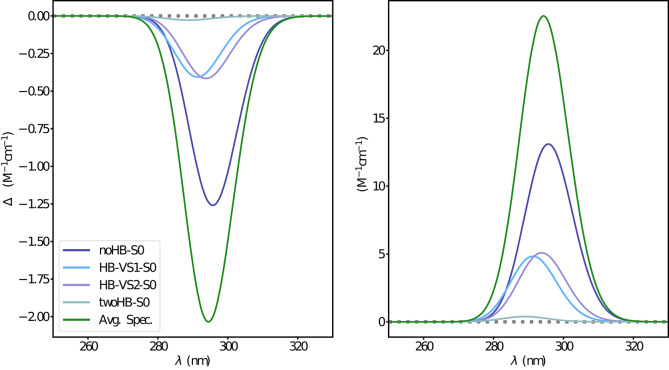
Electronic ECD **(left)** and OPA **(right)** spectra of camphor in methanol obtained through the ONIOM/EE-PMM method. The different contributions from each cluster are shown.

**Figure 8:**
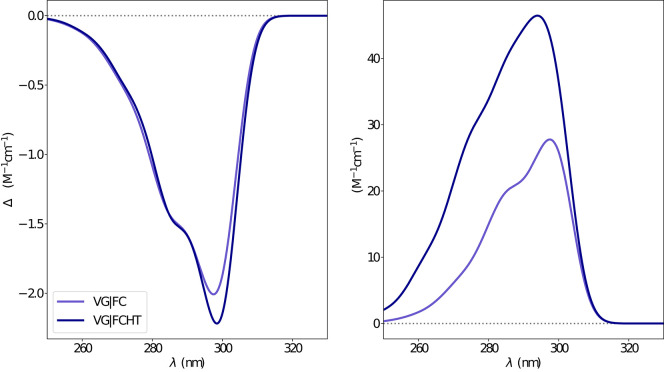
Vibronic ECD **(left)** and OPA **(right)** spectra of camphor in methanol as obtained through VG|FC and VG|FCHT computational procedures in conjunction with PCM model to treat the environment effect.

**Figure 9:**
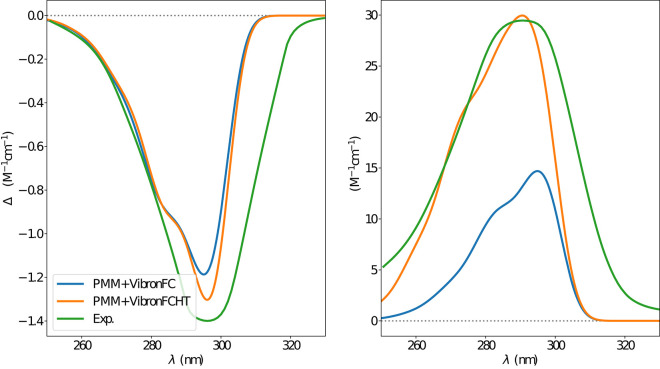
ECD **(left)** and OPA **(right)** spectra of camphor in methanol as obtained by combining the ONIOM/EE-PMM procedure outcome with VG|FC (blue line) and VG|FCHT (orange line) models to simulate the vibronic coupling, the corresponding experimental spectrum is also reported (green line). Experimental spectra taken from Longhi et al. (2016).

The first step was the simulation of the absorption spectra. The experimental results show an isolated well-defined band for both OPA and ECD spectra without any apparent vibronic sub-structure. According to Longhi et al. (2013), these results suggest a non-negligible interaction with the solvent molecules. In Figure 7, the electronic absorption spectra obtained at the ONIOM/EE-PMM level are reported. Indeed, the four clusters lead to quite different spectra both in terms of energy and intensity, thus contributing to the overall asymmetric shape of both OPA and ECD spectra (see Figure S3 for unweighted cluster spectra). Vibrational modulation effects bring an additional contribution to the final spectra and the corresponding results including bulk solvent effect by mean of the PCM are reported in Figure 8. It is noteworthy that HT terms smooth the prominence of the first band and, especially for the OPA spectrum, cause a non-negligible intensity gain. Finally, as reported in Figure 9, a proper account of specific solvent effects by means of the overall ONIOM/EE-PMM procedure modulates the HT contributions and, in turn, improves the overall agreement with the experimental spectrum, as already observed in the case of the related camphorquinone molecule (Del Galdo et al., 2020).

After having tuned the procedure on absorption spectra we analyzed the corresponding emission spectra. As discussed in section 3.1, the excited electronic state must be described in terms of two disjoint conformers, whose relative stability is a key parameter in the reproduction of the overall spectra. Relying on previous investigations (Barone et al., 2015; Fusè et al., 2019; Paoloni et al., 2020), we employed in this case the B2PLYP-D3 level of theory also accounting for bulk solvent effects by means of the PCM approximation. The results are reported in Table 1, and ΔG PCM values were used to compute Boltzmann population at 300 K in order to get the final simulated spectra.

**Figure 10:**
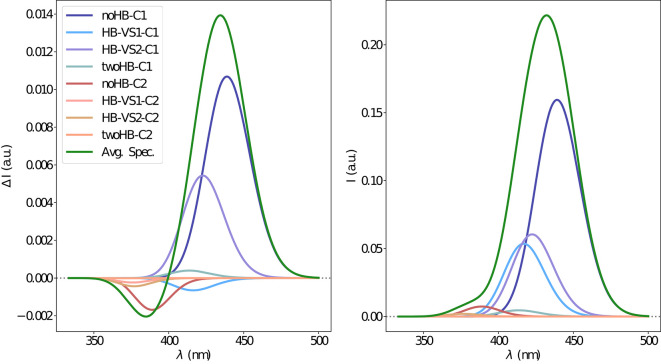
Electronic CPL **(left)** and OPE **(right)** spectra of camphor in methanol obtained through the ONIOM/EE-PMM method. The different contributions from each cluster for each excited state simulations are shown.

**Figure 11:**
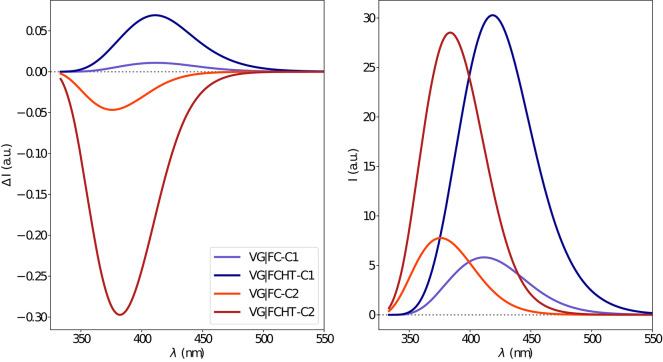
Vibronic CPL **(left)** and OPE **(right)** spectra of camphor in methanol as obtained through VG|FC and VG|FCHT computational procedures in conjunction with PCM model to treat the environment effect.

**Figure 12:**
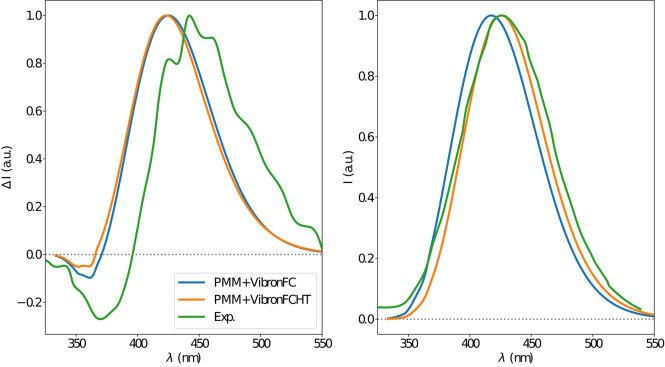
CPL **(left)** and OPE **(right)** spectra of camphor in methanol as obtained by combining the ONIOM/EE-PMM procedure outcome with VG|FC (blue line) and VG|FCHT (orange line) models to simulate the vibronic coupling, the corresponding experimental spectrum is also reported (green line). Experimental spectra taken from Longhi et al. (2016).

The electronic emission spectra obtained through application of the ONIOM/EE-PMM method are reported in Figure 10. The differential contributions of both camphor conformers and, for each of them, of the different clusters, are clearly shown. In particular, the presence of different numbers of solute-solvent H-bonds led to quite asymmetrical spectra since these interactions shift the spectrum toward higher energies. On the other hand, the equilibrium between the two main solute conformers is responsible for the inversion of the sign of the transition. In fact, already at the ONIOM/EE-PMM level the bisignate nature of the CPL is well-reproduced. This clearly highlights the relevance of both proper sampling of the solvent distribution and proper weighting of conformer populations. Similarly to the absorption case, the HT term substantially affects the transition intensity. In Figure 11, the OPE and CPL vibronic spectra are reported. It is quite apparent that the CPL intensity of the less populated conformer is higher than that of the prevailing one and it partially compensates the lower population at 300 K. The result of combining vibronic and ONIOM/EE-PMM contributions in the emission spectra is shown in Figure 12. The results are in good agreement with the experimental ones, and it is worth recalling at this point that the full computational procedure does not involve any empirical correction. Moreover, it should be noted that the original CPL spectra from the work of Longhi et al. (2013) are quite noisy (the authors claimed that for both absorption and emission solute-solvent interactions are responsible for the quenching of the vibronic features) and the structure of the experimental CPL signal in Figure 12 is probably due to digitization artifacts. Comparison of the simulated spectra with their experimental counterparts shows that the OPE band-shape is well reproduced by our approach, whereas the agreement is only fair for the CPL spectrum. One of the possible limitations of our computational protocol is the approximate VG vibronic model, which could be too rough to catch and accurately describe the contribution of the vibration on the spectra. Although more refined models could possibly lead to more accurate results, our integrated approach is capable to improve the final results of both ONIOM/EE-PMM and vibronic models taken individually, providing a simulated CPL spectrum significantly closer to the experimental one without any huge increase of the computational cost.

## 4. Conclusions

The main aim of the present contribution was the development of a cheap yet accurate multi-scale strategy for the study of solvatochromic shifts of medium-size flexible chromophores in condensed phases. Explicit treatment of a large amplitude motion and clustering of different MD simulations for each stationary point along the LAM allow the extension of our previous approach from semi-rigid to flexible molecules. At the same time, fitting of atomic charges for excited electronic states permits their MD simulation and the following computation of emission spectra. As a test case, we have chosen a flexible system characterized by remarkable chiroptical properties (namely, the camphor dye in methanol solution), which requires, inter alia, the challenging reproduction of significantly different ECD and CPL spectra. The computational strategy starts from the collection of transition energies and dipole moments tuned by environmental effects provided by MD simulations. Next, vibrational modulation effects on the different electronic transitions are taken into account by a fully quantum mechanical approach including Franck-Condon and Herzberg-Teller contributions together with bulk solvent effects described by a polarizable continuum model. At variance with previous applications, a large amplitude motion has been explicitly considered by means of a discrete-variational approach. Specific chromophore-environment interactions and their fluctuations are described by a recently proposed hybrid procedure, which combines a variational approach (ONIOM/EE), with the PMM perturbative approach. In this way, it becomes possible to obtain accurate results without the need to perform explicit computations for a statistically significant number of solute-solvent configurations. For each stationary point of this flexible system (i.e., the two energy minima of the S1 electronic state and the transition state ruling their inter-conversion corresponding to the single minimum of the ground electronic state) we partitioned the overall simulations into four subsets (clusters) related to the presence or not of strong solute-solvent hydrogen bonds. Although the flexibility of the system increases the number of explicit QM computations with respect to semi-rigid chromophores, the proposed ONIOM/EE-PMM approach strongly reduces the computational effort with respect to a standard QM/MM computation. The good agreement between all the computational results and their experimental counterparts, gives confidence about the robustness and reliability of the overall procedure and permits the analysis of the role of different contributions (stereo-electronic, dynamic, environmental) in tuning the overall spectra.

## Data Availability Statement

The original contributions presented in the study are included in the article/Supplementary Material, further inquiries can be directed to the corresponding authors.

## Author Contributions

All authors listed have made a substantial, direct and intellectual contribution to the work, and approved it for publication.

## Conflict of Interest

The authors declare that the research was conducted in the absence of any commercial or financial relationships that could be construed as a potential conflict of interest. The reviewer LE declared a past co-authorship with one of the authors VB to the handling editor.
