# Supplementary material for: CPL Spectra of Camphor Derivatives in Solution by an Integrated QM/MD Approach
Source: Front Chem. 2020 Jul 7;8:584. doi: 10.3389/fchem.2020.00584 (PMC7358700; doi:10.3389/fchem.2020.00584)
Supplement: Supplementary file 1 [file Data_Sheet_1.PDF]

Supplementary Material

1 GROUND STATE MOLECULAR DYNAMICS RESULTS

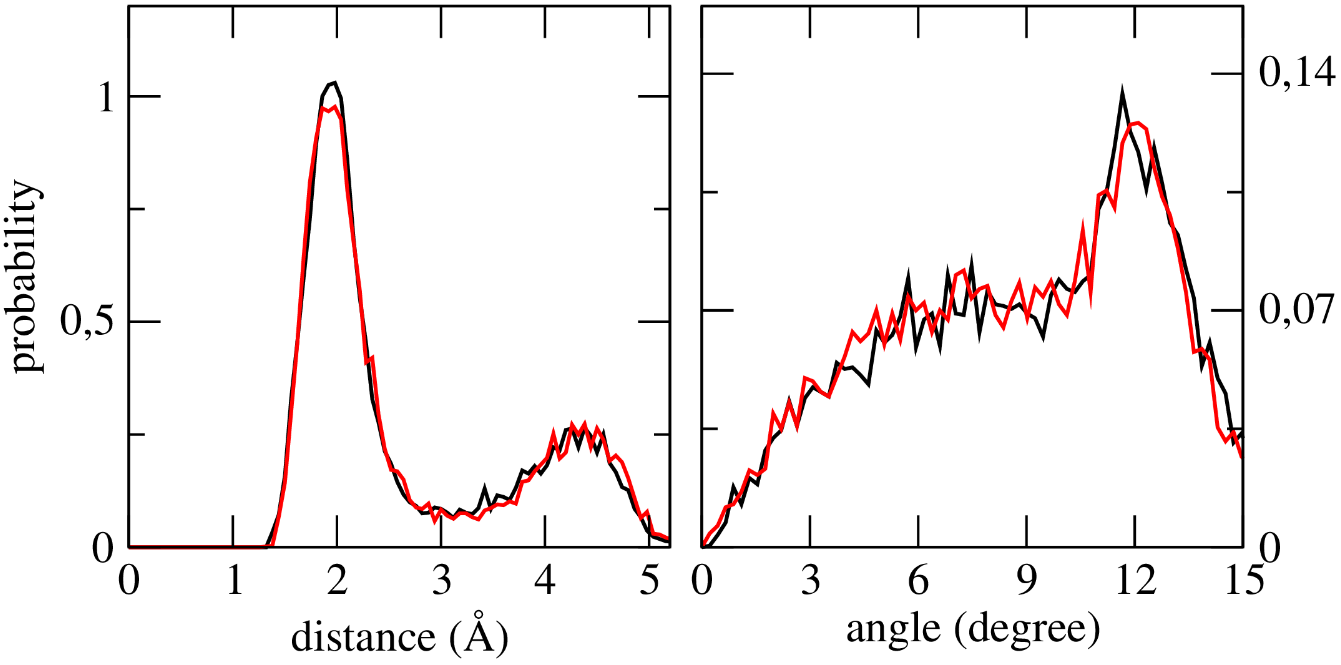

Figure S1. Distribution functions of each VS - (methanol hydroxyl) H distance (left panels) and of each VS- (methanol hydroxyl) H - (methanol hydroxyl) O angles (right panels) as obtained from the ground state simulation of Camphor in methanol. Data computed for the first and second VSs are shown as black and red lines, respectively

	Population
noHB	59.04
HB-VS1	18.83
HB-VS2	20.76
twoHB	1.37

Table S1. Population per cent of the clusters within the ground state simulation of Camphor in methanol.

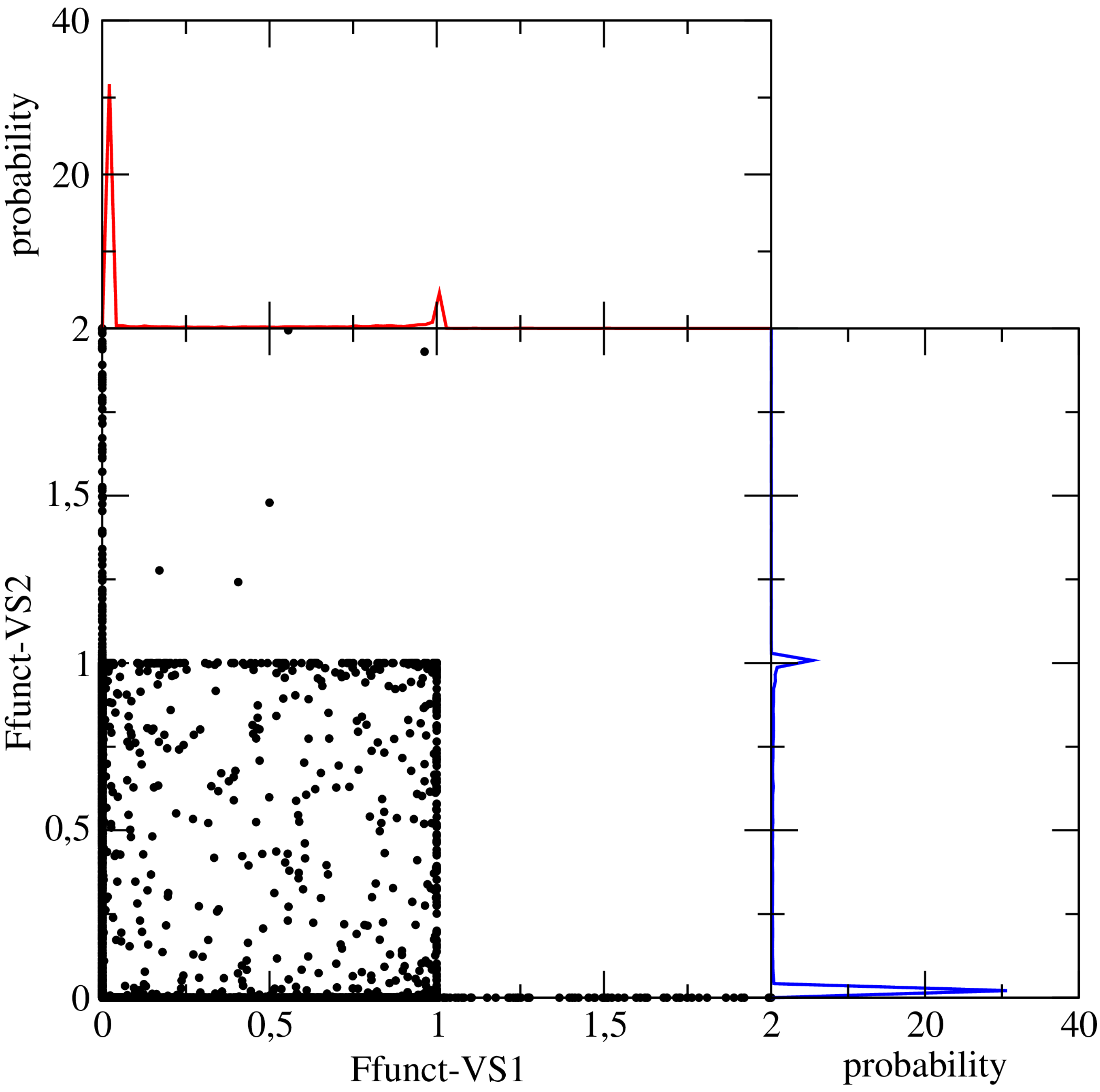

Figure S2. Space defined by the values of the F function computed for each VS in the camphor molecule from the ground state simulation. The corresponding probability distributions of each F function are also shown.

2 ONIOM/EE-PMM SPECTRA

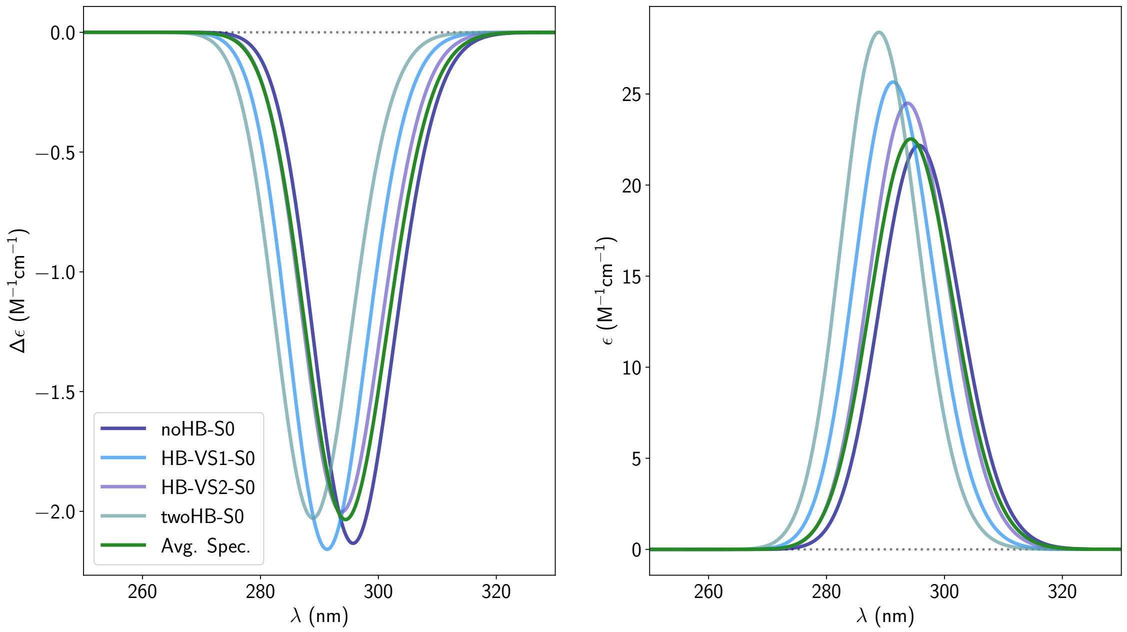

Figure S3. Electronic ECD (left panel) and OPA (right panel) spectra of camphor in methanol obtained through the ONIOM/EE-PMM method. The unweighted spectra of each cluster are shown.

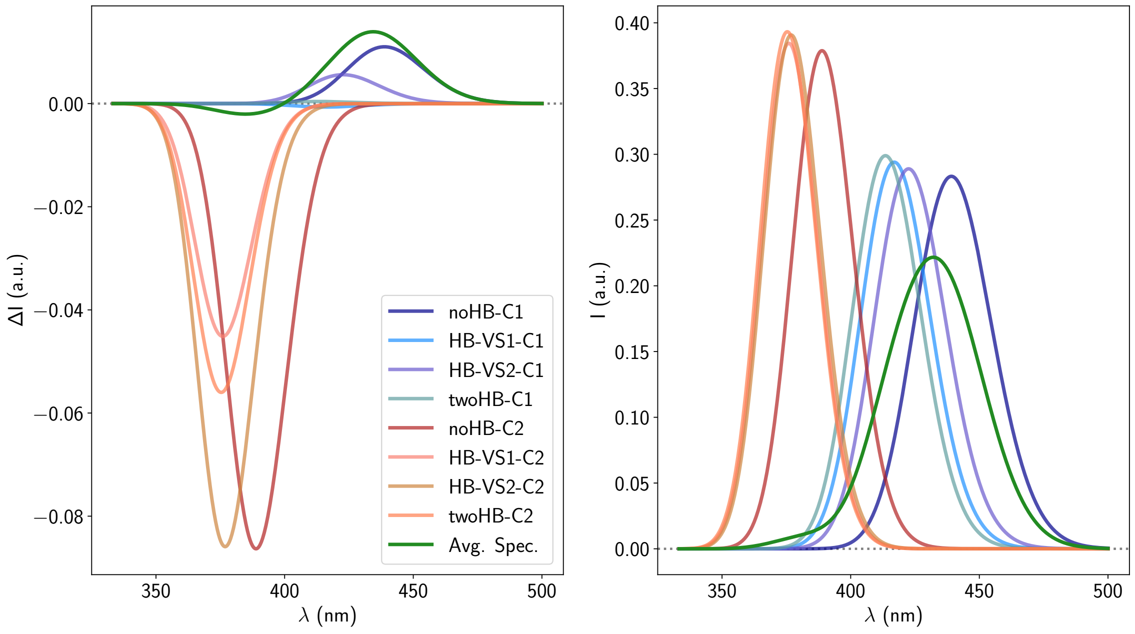

Figure S4. Electronic CPL (left panel) and OPE (right panel) spectra of camphor in methanol obtained through the ONIOM/EE-PMM method. The unweighted spectra of each cluster are shown.
